# Rare Case of Neuroendocrine Metastasis to the Left Ventricle

**DOI:** 10.1016/j.jaccas.2024.102399

**Published:** 2024-06-14

**Authors:** Said Hajouli, Jacob A. Misenheimer, Amir Kamran, Kerry Drabish, Frank H. Annie, Fahad F. Bafakih, Yodsui F. Hernandez, Ahmad Elashery

**Affiliations:** a Cardiovascular Division, Charleson Area Medical Center, Charleston, West Virgina, USA; b Hematology-Oncology, Charleston Area Medical Center, Charleston, West Virgina, USA; c Pathology Department, Charleston Area Medical Center, Charleston, West Virgina, USA

**Keywords:** carcinoid, carcinoid heart disease, metastasis, neuroendocrine tumor

## Abstract

Carcinoid syndrome is a constellation of signs and symptoms caused by different hormones produced by carcinoid tumors. Very rarely, those tumors can metastasize to the heart and cause cardiac involvement of the tumor. This study presents a very rare case of secondary cardiac tumor affecting the left ventricle from a metastatic carcinoid tumor originating from the small intestine without carcinoid valvular heart disease.

## Graphical Abstract

**Figure:**
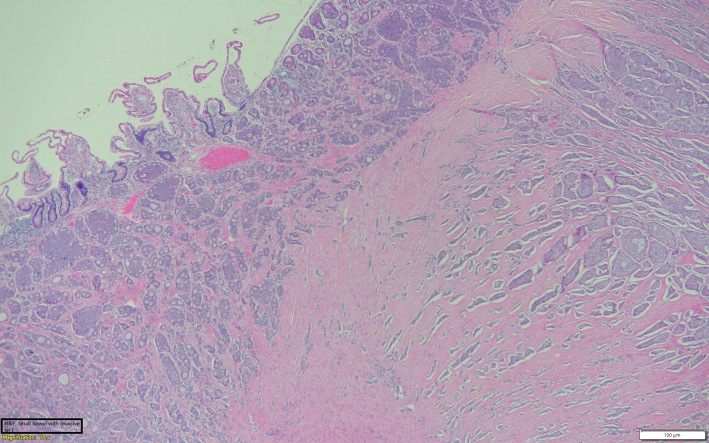

## History of Presentation

A 53-year-old man presented to the hospital in September 2022 with a 1-day history of sudden onset of left lower quadrant (LLQ) crampy abdominal pain and diarrhea. Pain was intermittent (5 of 10), postprandial, and associated with vomiting and diarrhea 1 to 2 times per day with no radiation, hematochezia, or hematemesis. His vitals were as follows: temperature 36.4 °C, heart rate 108 beats/min, blood pressure 147/87 mm Hg, and respiratory rate 17 breaths/min. His physical examination revealed severe LLQ abdominal tenderness without rebound.

**Learning Objectives**

• To be able to diagnose CHD if valvular regurgitation.
• To understand the role of Ga68 PET/CT and cardiac magnetic resonance for metastasis diagnosis.
• It is important to understand when to treat cardiac metastases which is only if patients have symptoms or RV failure.

## Past Medical History

The patient had a past medical history of diabetes mellitus controlled with metformin, hypertension controlled with lisinopril, hyperlipidemia, obstructive sleep apnea, and chronic kidney disease. His family history included 3 brothers and his father who had colon cancer in their 50s.

## Differential Diagnosis

Because the patient had pain in the LLQ, the differential diagnosis included acute sigmoid diverticulitis, colonic perforation, ischemic colitis, and renal colic/ureterolithiasis.

## Investigations

Abdomen/pelvis computed tomography showed nephrolithiasis and an incidental finding of a mesenteric mass. His 24-hour urine 5-hydroxyindoleacetic acid level was 23.5 mg/24 h (normal <9.4). He was referred for surgery evaluation.

## Management

**Figure 1:**
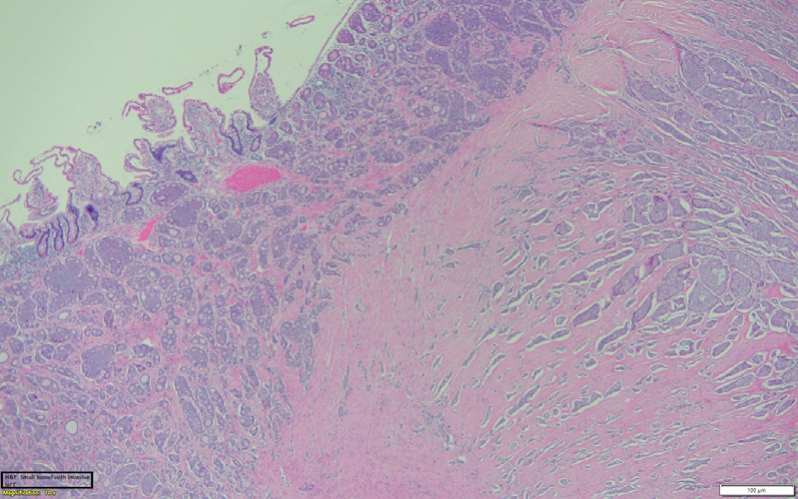
Tissue Sample Showing the Neuroendocrine Tumor

**Figure 2:**
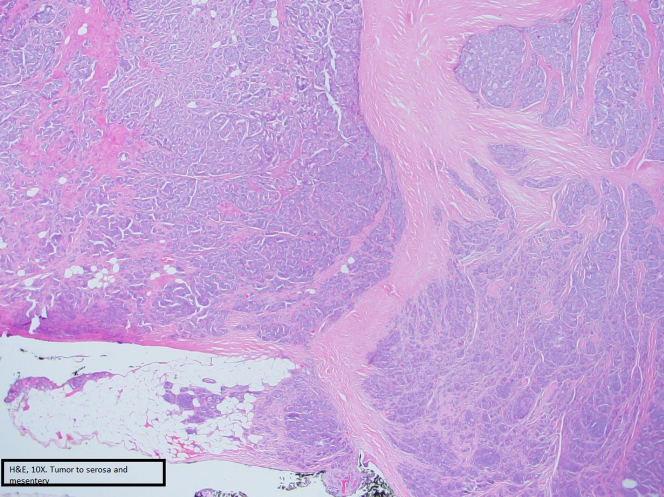
Tissue Sample Showing the Neuroendocrine Tumor

**Figure 3:**
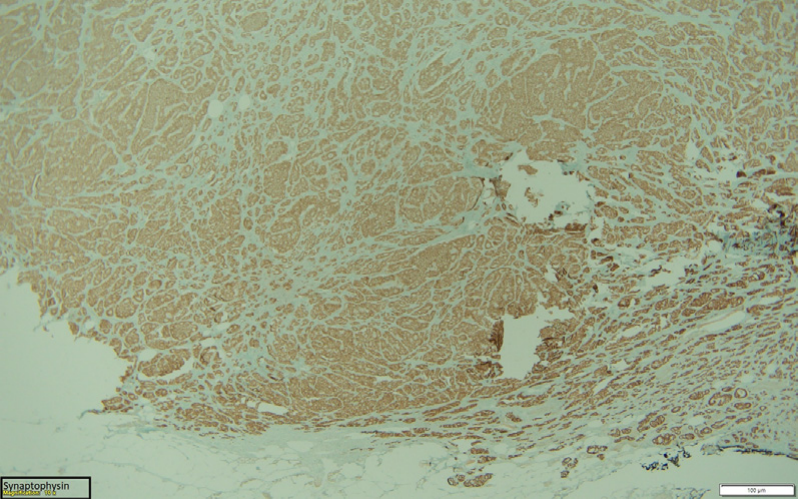
Tissue Sample Showing the Neuroendocrine Tumor

In December 2022, he underwent an exploratory laparotomy. He had a palpable 2- × 2-cm unifocal well-differentiated grade 1 small intestine mass invading the visceral peritoneum. He had palpable lymphadenopathy along the base of the mesentery that was not resected completely. The mesenteric mass likely represents lymphatic spread to a regional lymph node. The primary tumor with 3 lymph nodes was resected. Multiple palpable lymph nodes were left in situ, representing an important focus for continuing surveillance. Pathology showed a well-differentiated neuroendocrine tumor (NET) without lymphovascular/perineural invasion (Figure 1, Figure 2, Figure 3).

**Figure 4:**
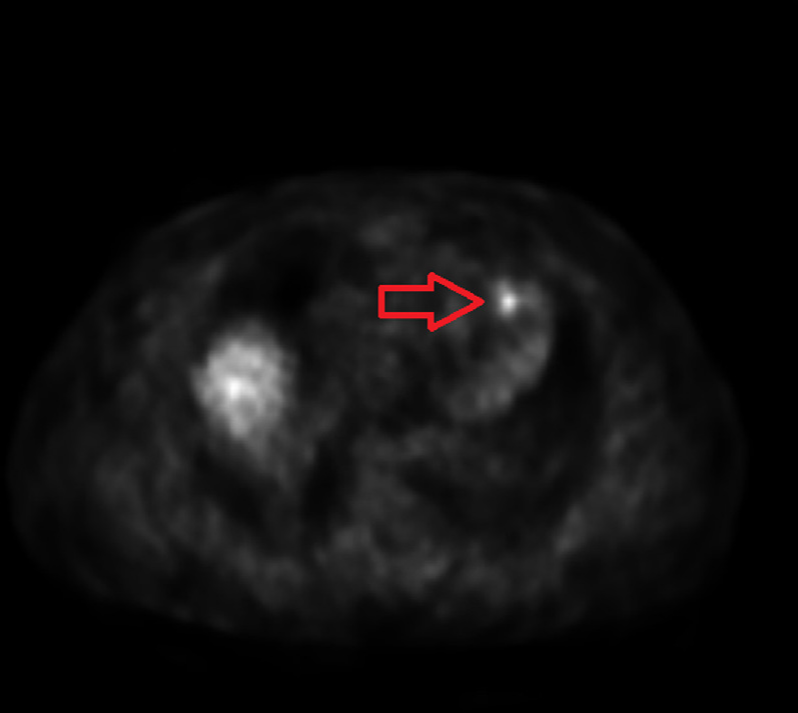
Gallium-68 Positron Emission Tomography/Computed Tomography Scan Gallium-68 positron emission tomography/computed tomography scan showing a small focus of uptake (arrow) near the left ventricular myocardium apex.

**Figure 5:**
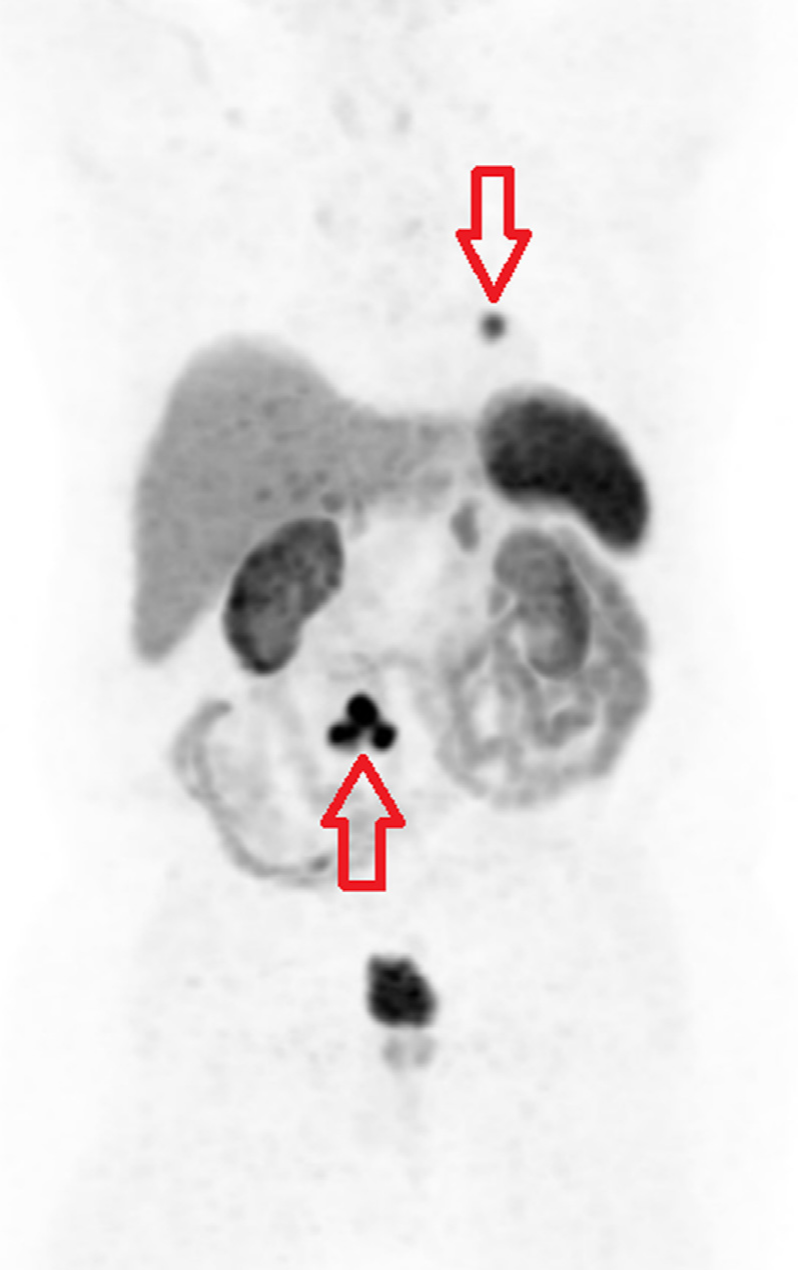
Gallium-68 Positron Emission Tomography/Computed Tomography Scan Gallium-68 positron emission tomography/computed tomography scan showing the uptake in the mesenteric mass and left ventricular apex (arrows).

**Figure 6:**
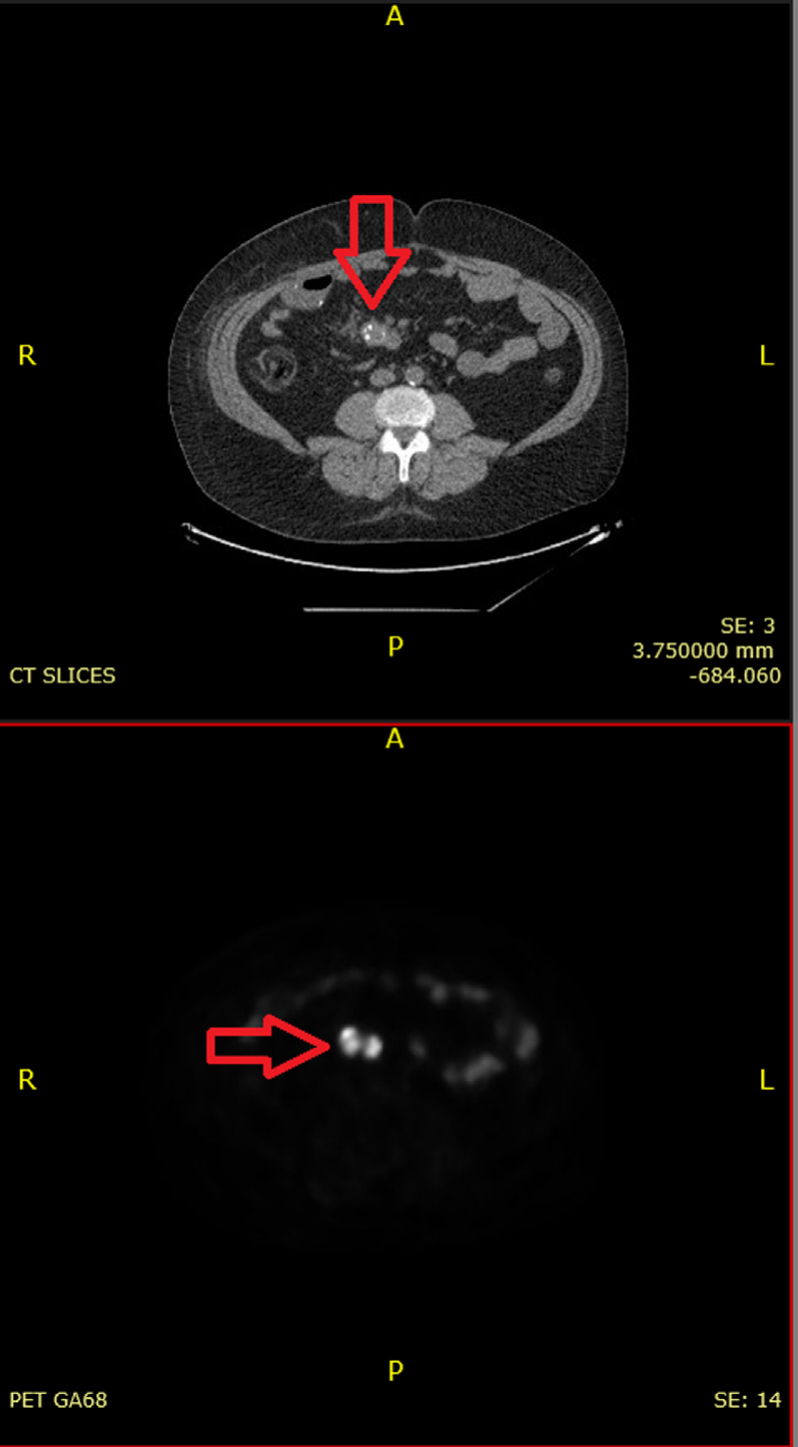
Gallium-68 Positron Emission Tomography/Computed Tomography Scan Gallium-68 positron emission tomography/computed tomography scan showing intense pathologic uptake in the mesenteric mass (arrow).

**Figure 7:**
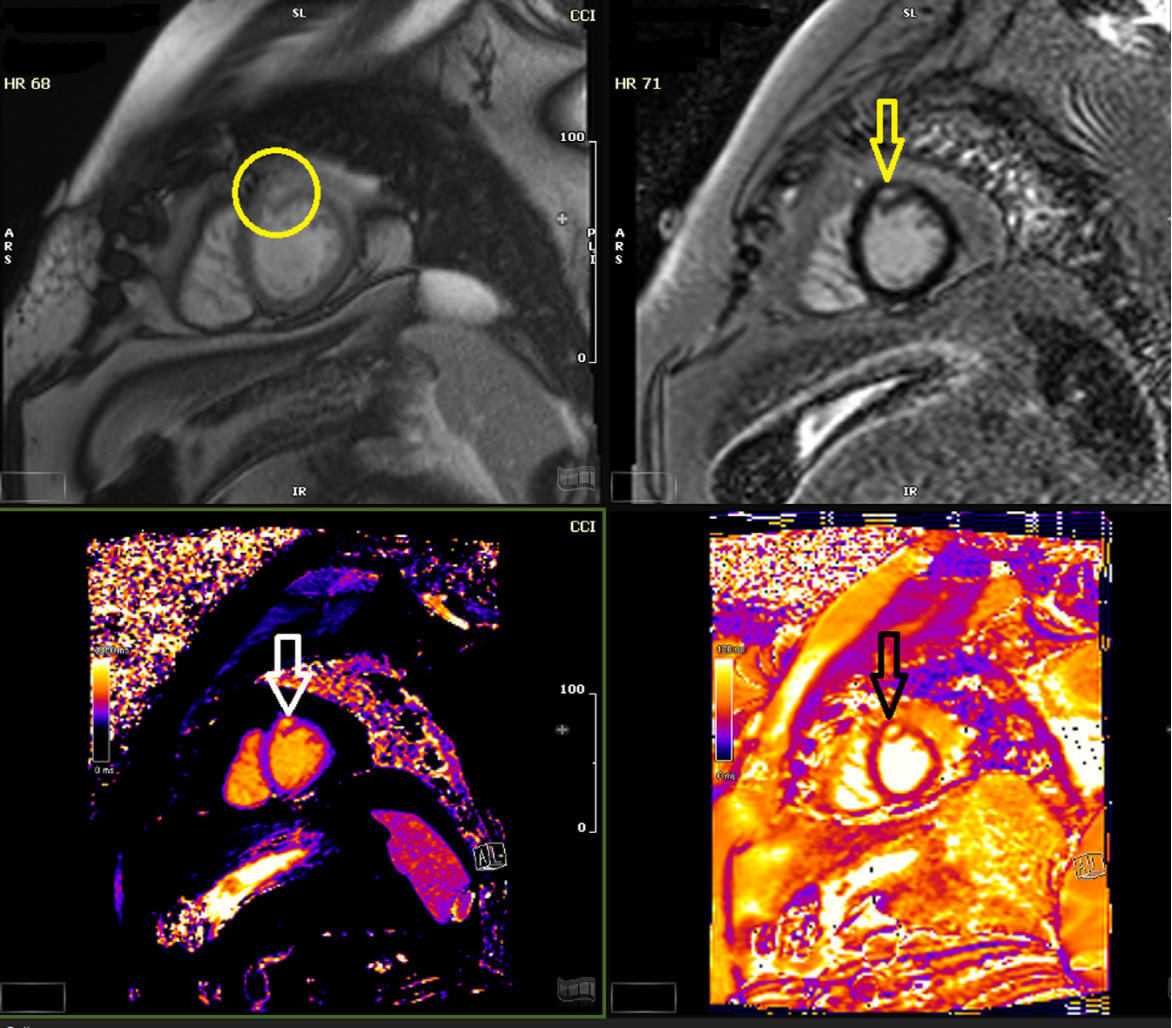
Cardiac Magnetic Resonance Cine steady-state free precession sequences, late gadolinium enhancement, T1, T2, showing distal anterior wall of late gadolinium enhancement (arrows/circle).

His diarrhea improved after surgery but did not resolve completely. Leaving the mesenteric mass in situ is the likely source of continued symptom-related serotonin and other vasoactive amines. Given his persistent symptoms, a Gallium-68 (Ga68) positron emission tomography (PET) scan was performed to rule out metastasis. It showed intense pathologic uptake in a mesenteric mass most consistent with NET and a small focus of nonspecific cardiac uptake within the left ventricular (LV) myocardium near the apex (Figure 4, Figure 5, Figure 6). He was diagnosed with carcinoid syndrome and started on lanreotide 120 mg every 4 weeks. Due to the uptake in the heart, he had transthoracic echocardiogram (TTE) which showed normal LV wall thickness and systolic function (ejection fraction 55%-60%) with no valvular disease. Due to the cardiac uptake on the PET scan, he had cardiac magnetic resonance which showed a 5- × 9-mm small area of late gadolinium enhancement in the distal LV anterior wall that was also hyperintense in T1 and T2 mapping (Figure 7). When compared with PET/CT, it was the same area of hyperactivity in Ga68 PET. In the presence of systemic NET, this was diagnosed as cardiac involvement with a carcinoid tumor metastasis, which was most likely a hematologic spread of the native gastrointestinal NET.

## Discussion

The most common cardiac tumors are metastases from noncarcinoid primary tumors. Most primary cardiac tumors are benign with only 10% being malignant. Myxoid tumors represent approximately 50% of all primary cardiac tumors. Almost all NETs are considered malignant. There are just differences in how aggressive they are. Carcinoids are rare tumors accounting for 2.5 to 5 per 100,000 per year.^1^ NETs can affect the heart by metastasizing to the liver and secreting high concentrations of vasoactive amines, especially serotonin which, by stimulating serotonin receptors on the right heart valves, causes fibroblast proliferation (scarring). Carcinoid heart disease (CHD) can take different manifestations including valvular disease, coronary artery vasospasms, arrhythmias, and direct myocardium disease from metastases. Valvular heart disease is a common CHD finding and is usually found in the right side of the heart (tricuspid and pulmonic valves). Fibrous tissue deposition in the endocardium is a pathologic finding of the CHD and occurs on the ventricular aspect of the tricuspid valve and the arterial aspect of the pulmonic valve. Due to inactivation of the humoral products by the lungs, the left side of the heart is usually spared. However, if there is a patent foramen ovale with right-to-left shunt at the atrial level, left-sided heart valves can be affected (in 5%-10% of CHD cases). Occasionally, the left side can be affected without a right-to-left shunt, as in cases of severe uncontrolled carcinoid syndrome (with very high serotonin levels) or in cases of bronchial carcinoid tumors. This affects the valves instead of the cardiac chambers. Right-sided valvular disease in the absence of left-sided valvular disease should raise a concern and prompt screening for carcinoid syndrome.

CHD is rarely due to a cardiac metastasis from a primary carcinoid tumor, typically from the small intestine. This direct metastasis to the heart can be found in 2% to 4% of metastatic NETs;^2^ 73% of cardiac metastases have been associated with serotonin-related fibrotic carcinoid valve disease.^3^ Metastatic NET is usually to the liver, lymph nodes, and bones; intracardiac metastasis is not common and can occur in 0.7%.^4^ Very rarely, NETs originate from the heart as the only NET in the body.^5^

Screening patients with NETs for cardiac metastases and CHD is recommended every 6 to 12 months. N-terminal pro–B-type natriuretic peptide is a sensitive test to rule out CHD^6^; if this value is >260 ng/mL, a TTE is recommended to evaluate for valvular disease or metastases. However, lesions <1 cm might be missed by TTE. Most of the well-differentiated NETs (eg, carcinoid tumors) express somatostatin receptors that can be detected by radiolabeled tracers used in PET/CT scans. These can include Ga-68 DOTATATE, Ga-68 DOTATOC, and Cu-64 DOTATATE and are crucial in diagnosing even small lesions.^7^ Ga-68 DOTATATE PET scanning has the greatest sensitivity to detect metastases from NETs and is the preferred test for this use in accord with the North American Neuroendocrine Tumor Society guidelines. Ga-68 DOTATATE has affinity for the somatostatin receptor–positive NETs and has high sensitivity and specificity to localize NET lesions.^8^ It gives important information about the tumor cell receptors status and helps staging, restaging, and target therapy. If a cardiac focus of uptake is detected by Ga-68 PET scan, further assessment with cardiac magnetic resonance is indicated. F-18-dihydroxy-phenylalanine PET scan can also be used to screen for cardiac metastases from NETs. The somatostatin receptor scintigraphy and TTE can diagnose the cardiac metastases but cannot delineate the surrounding tissues. Therefore, cardiac magnetic resonance is valuable to yield the tumor size, location, borders, and infiltration and to determine the connection to the myocardium or the presence of pericardial effusion. Magnetic resonance imaging can also be used to assess the vascularity of the tumor and the presence of fibrosis. The anatomic and mobility characteristics of the tumors can be identified with the short and long axis cine steady-state free precession sequence magnetic resonance imaging. First pass perfusion magnetic resonance imaging is the standard rest perfusion sequence that helps determine the vascularity of the tumor.

Cardiac metastases from NETs are usually asymptomatic but can cause heart failure symptoms, arrhythmias, cardiac obstruction, or cardiac arrest. They are usually incidental findings by PET scan. Asymptomatic cardiac metastases might not require additional treatment other than treating the underlying primary tumor with surgery, radiofrequency ablation, somatostatin analogs, or chemotherapy. Surgical removal of the cardiac lesions can be considered in patients with arrhythmia or cardiac obstruction from mass effect. However, such surgeries might be too dangerous, and discussion with patients about the risks and benefits is very important. Radiation therapy is an option in some cases.^9^ The growth and effects of the cardiac metastases are uncertain, and cardiac lesions might remain stable even without treatment. Treatment for valvular carcinoid disease (serotonin-induced fibrosis) is surgery when cardiac symptoms (eg, right ventricular failure/enlargement, right ventricular systolic dysfunction) develop.

The present case is unique because the patient did not have liver metastases from the NET; however, he had cardiac metastasis. The cardiac metastasis was the only evidence of this patient’s gastrointestinal NET distant hematogenous spread. The mesenteric mass likely represents spread to a regional lymph node; it is very rare to only have cardiac metastasis. In addition, despite having carcinoid syndrome symptoms, he did not have the typical cardiac valvular involvement. This is due to the absence of liver metastases, which often provide the source of tumor secreting a high concentration of serotonin, which stimulates serotonin receptors on the inner lining of the heart, including the valves. Serotonin (carried largely within platelets), is deactivated within the lungs, thereby sparing the left heart valves.

## Follow-Up

His diarrhea improved with lanreotide. He had a repeated Ga68 PET scan which showed stable hypermetabolic mesenteric abdomen mass and focal focus of gallium uptake at the left side heart, and there was no new disease detected.

## Conclusions

We present a very rare case of metastatic cardiac tumor involving the left ventricle and originating from a primary carcinoid tumor of the small intestine, without carcinoid valvular heart disease.

## Funding Support and Author Disclosures

The authors have reported that they have no relationships relevant to the contents of this paper to disclose.
